# Wound healing properties of *Copaifera paupera* in diabetic mice

**DOI:** 10.1371/journal.pone.0187380

**Published:** 2017-10-31

**Authors:** Jorge Luis Amorim, Janaína de Barros Figueiredo, Ana Claudia Fernandes Amaral, Eliane Gouvêa de Oliveira Barros, Célia Palmero, Maria Athana MPalantinos, Aline de Souza Ramos, José Luiz Pinto Ferreira, Jefferson Rocha de Andrade Silva, Claudia Farias Benjamim, Silvia Luciane Basso, Luiz Eurico Nasciutti, Patricia Dias Fernandes

**Affiliations:** 1 Universidade Federal do Rio de Janeiro, Instituto de Ciências Biomédicas, Laboratório de Farmacologia da Dor e da Inflamação, Rio de Janeiro, Brasil; 2 Universidade Federal do Rio de Janeiro, Instituto de Biofísica Carlos Chagas Filho, Rio de Janeiro, Brasil; 3 FIOCRUZ, Farmanguinhos, Rio de Janeiro, Brasil; 4 Universidade Federal do Rio de Janeiro, Instituto de Ciências Biomédicas, Laboratório de Interações Celulares, Rio de Janeiro, Brasil; 5 Universidade Federal do Amazonas, Departamento de Química, Laboratório de Cromatografia, Manaus, Brasil; IDI, Istituto Dermopatico dell'Immacolata, ITALY

## Abstract

Copaifera oleoresin is one of the most used natural products in popular medicine all over the world. Among other effects (i.e., anti-inflammatory, antinociceptive, microbicidal) one of the most well-known is its wound healing capacity. However, the mechanism by which the oleoresin presents its effect is still not clear. In this study, our aim was to evaluate the wound healing capacity of oleoresin obtained from *Copaifera paupera*, its mechanism of action and identify its major components. For these purposes, diabetic Swiss *Webster* mice were topically treated with oleoresin (100, 150 or 200 mg/kg) for 14 consecutive days after an excision was performed in the back of the mice. Cytokines, wound retraction and histological evaluation were conducted at 3, 7 and 10 days (for cytokines); 0, 3, 7, 10 and 14 days (for wound retraction); and 7 and 14 days (for histological evaluation). Our data indicate that oleoresin significantly reduced production of MCP-1 and TNF-α at days 7 and 10 post-excision and increased IL-10 production at both days. All treatments demonstrated an effect similar or higher to that in collagenase-treated mice. Histological evaluations demonstrated that higher dose treatment resulted in better resolution and closure of the wound and higher levels of collagen deposition and indexes of re-epithelialization even when compared with the collagenase-treated group. The treatment with oleoresin from *Copaifera paupera* demonstrated that it is even better than an ointment routinely used for improvement of wound healing, suggesting this oleoresin as an option for use in diabetic patients.

## Introduction

Almost all multicellular organisms present a universal phenomenon of tissue repair. Wound healing is a complex event involving different cell types, primarily keratinocytes, fibroblasts, endothelial cells of vessels and recruited immune cells, and their associated extracellular matrix. In healthy individuals, this process is highly efficient. However, when the normal repair response goes wrong, there are two major outcomes: either an ulcerative skin defect (chronic wound) or excessive formation of scar tissue (hypertrophic scar or keloid) [1]. In some chronic diseases (i.e., diabetic foot ulcers, venous leg ulcers and pressure ulcers), there is some heavy inflammatory infiltrate, with cells phenotypically different from their equivalents in a healing acute wound, leading to difficulties in resolution and closure of wounds [2]. In this regard, the search for new treatments or drugs that could improve healing in diabetic patients continues to be a goal in medicine.

Copaiba oleoresin is a popular name assigned to the oleoresin obtained from the trunk of various species of *Copaifera* (Fabaceae) and is one of the most important renewable sources of natural remedies for different populations [1]. This plant's resinous derivative has been used by Indians from the Northern and Northeastern parts of Brazil, especially in Amazonas State, for its anti-inflammatory and antinociceptive effects, among others [3, 4].

In addition to its use in traditional medicine, studies have demonstrated that *Copaifera* oleoresin has several other effects, i.e., it can ameliorate the outcome of some inflammation-mediated diseases [4–6], develops antinociceptive activity [3, 4] and presents anti-tumoral effects [7].

However, until now, there is no work identifying and characterizing the mechanisms involved in the wound healing induced by *Copaifera* oleoresin topically administered into wounds. In this context, the aim of this work was to study the mechanisms of action of *Copaifera paupera* oleoresin in the wound healing process in diabetic mice.

## Materials and methods

### Animals

Male Swiss *Webster* mice (20–25 g) donated by Instituto Vital Brazil (Niteroi, Rio de Janeiro, Brazil) were used in this study. The animals were maintained in standard conditions (room with light-dark cycle of 12 h, 22 ± 2°C, from 60% to 80% humidity and food/water provided *ad libitum*). Animals were acclimatized to the laboratory conditions for at least 1 h before each test onset and were used only once throughout the experiments. Research was conducted in accordance with the internationally accepted principles for laboratory animal use and care as found in the European Community guidelines (EEC Directive of 1986; 86/609/EEC) and the US guidelines (NIH publication #85–23, revised in 1985). All protocols followed the principles and guidelines adopted by the National Council for the Control of Animal Experimentation (CONCEA), approved by the Biomedical Sciences Institute/UFRJ, Ethical Committee for Animal Research, with identification number DFBCICB015–04/16.

### Drugs and reagents

Ketamine and xylazine were purchased from Ceva Santé Animale (Brazil); alloxan, hematoxylin, eosin and Gomori stains were purchased from Sigma Aldrich (St. Louis, MO, U.S.A.); a protein assay kit was purchased from Pierce (BCA™ Protein Assay Kit, Pierce); cytokine kits were acquired from B&D (USA).

### Collection of oleoresin

Oleoresin from *Copaifera paupera* (Herzog) Dwyer was directly exudated from the trunk of a specimen located at Chico Mendes Reserve, Acre, Brazil, in July 2007. A voucher was deposited at the Herbarium of Goeldi Museum/Pará state (Number 35,524). The plant name has been checked with http://www.theplantlist.org.

### Gas Chromatography-Mass Spectrometry (GC/MS) analysis

The oleoresin was analyzed by GC/MS with electronic impact ionization on an Agilent 6890 system equipped with a DB-5MS fused capillary column (30 m x 0.25 mm; 0.25 μm film thickness) coupled to an Agilent 5973N selective mass detector (Agilent, Agilent Technology, CA, USA). Helium (1 ml/min) was used as carrier gas; the oven temperature program was 70–310°C at 5°C/min; split rate of 10:1; sample volume, 1 μl of the oil solution in hexane. Injector and detector temperatures were 290°C. Interpretation and identification of the fragmentation mass spectrum were carried out by comparison with the Wiley NBS mass spectrum database. The results were expressed as relative percentage of peak area in chromatogram.

### Preparation of oleoresin

*C*. *paupera* oleoresin (800 mg) was gently heated in a microwave, and the liquefied material was mixed with mineral oil (8 g) in a mortar and pestle for 10 min. The resulting liquid was used in the biological assays. The density of the oleoresin was measured and converted to mg with the aim of calculating the dose (in mg/kg) to be applied to wounds.

### Treatments

Mice were treated daily with *C*. *paupera* oleoresin (100, 150 or 200 mg/kg) at a final volume of 30 μl applied directly to each wound. After treatment, the mice were maintained in individual boxes for 30 minutes to allow total absorption of *C*. *paupera* oleoresin, after which they were returned to their cages. Treatments began at day zero immediately after wound excision. A negative control group was composed of mice treated locally with mineral oil. A positive control group was composed of mice treated locally with collagenase ointment (0.6 U/kg).

### Induction of diabetes

Mice received an intravenous injection of alloxan (65 mg/kg). After 7 days, an aliquot of blood was obtained from the tail vein, and blood glucose levels were evaluated in an automatic glucosimeter (One Touch^®^, Johnson & Johnson, Brazil). Animals with glucose levels higher than 350 mg/dl were considered diabetic and used in the experiments.

### Full-thickness excision wound model

A method developed by [8] with a few alterations was used to produce full-thickness excision wounds. Briefly, mice were anesthetized with ketamine (112.5 mg/kg)/xylazine (7.5 mg/kg), the dorsal surface was shaved and cleaned with ethanol 70%, and a full-thickness excision wound was made with a 10-mm punch biopsy. At different days according to the protocol, different procedures were performed on the wounds (see below).

### Macroscopic evaluation of the lesions

The lesions were photographed using a digital camera (Canon Powershot A2500 HD, 16 megapixels) maintained at a fixed distance of 9 cm from base, at days 0, 3, 7, 10 and 14. Photos obtained from 10 different animals per group were individually analyzed with the software ImageJ, and the area of each lesion was calculated. Percent contraction of area was calculated using the following formula:

%contraction=(((areainday0)–(areainanalyzedday))/areaofday0)x100.

### Histological procedures and cytokine quantification

At days 7 and 14 (for histology) or 3, 7 and 10 (for cytokine quantification), 10 mice per group and per day were sacrificed with an overdose of ketamine/xylazine, and tissue samples around the wound were collected. For histological procedures, samples were fixed in 10% buffered formalin (Sigma Aldrich, St Louis, MO) and embedded in paraffin. Then, 5-mm-thick slices were prepared for Hematoxylin & Eosin and Gomori´s Trichrome stains. Histological images were obtained in an optical microscopic of conventional light (Nikon Eclipse E400) with an Evolution^TM^VF Color Cooled-Media Cybernetics digital camera and analyzed in QCapture software.

For cytokine quantification, tissue samples were mixed with a protease inhibitor cocktail (Roche, Germany) and triturated in an ultraturrex at 12,000 rpm, 10 min, 4°C. IL-1β, IL-6, IL-10, IFN-γ, TNF-α, and MCP-1 were quantified by enzyme-linked immunosorbent assay (ELISA) according to the manufacturer’s instructions (B&D, USA).

### Protein quantification

Supernatants obtained from wound homogenates obtained from 10 mice per group were analyzed using the BCA method (BCA™ Protein Assay Kit, Pierce) following the manufacturer’s protocol, and the amount of protein in each sample was determined in a microplate reader (FlexStation 3, Molecular Devices, EUA) at 570 nm.

### Histological analyses and index calculation

Tissue samples were analyzed as described by [9] with minor modifications and according to the following histological criteria: extension of re-epithelialization, maturation and organization of the epidermis, collagen deposition and infiltration of inflammatory cells. Tissues were observed and evaluated using a 0–3 rating scale into four categories (epithelialization, vascularization, inflammatory cells and collagen deposition response). For each group, 4 different slides were independently analyzed. From each slide, 10 different areas were analyzed. All procedures were performed by two independent persons in a double-blind procedure.

### Statistical analysis

Each experimental group consisted of 10 mice. The results were presented as the mean ± S.D. calculated using Prism Software 5.0 (GraphPad Software, La Jolla, CA, USA). Statistical significance between groups was determined using the application of two-way analysis of variance (ANOVA) followed by Bonferroni's test. P values less than 0.05 were considered to be significant.

## Results

The composition of *C*. *paupera* oleoresin was determined by GC-MS analysis (Table 1). The sesquiterpene α-copaene was the major component, representing 22.9%, followed by the diterpenes hardwickiic acid (8.1%) and kaur-16-ene (6.5%). The identified sesquiterpenes and diterpenes of the oleoresin corresponded to 60.2 and 25.4%, respectively.

**Table 1:** Volatile constituents (%) of *Copaifera paupera* oleoresin.

Compounds	RI	Composition (%)
α-cubebene	1351	5.7
α-muurolene	1499	0.2
α-copaene	1378	22.9
β-cubebene	1390	3.7
*trans*-caryophyllene	1423	5.5
α-bergamotene	1434	0.5
(+)-aromadendrene	1442	0.3
α-humulene	1454	0.9
γ-selinene	1455	1.0
Alloaromadendrene	1461	2.7
germacrene D	1480	0.5
α-amorphene	1485	1.0
β-selinene	1489	0.5
γ-gurjunene	1473	0.2
epi-cubebol	1493	0.5
*trans*-β-guaiene	1500	0.7
*cis*-calamenene	1521	0.6
δ-cadinene	1524	7.2
cadina-1,4-diene	1532	0.2
α-calacorene	1544	0.3
β-calacorene	1564	0.2
*d*-ledol	1565	0.3
Spathulenol	1576	0.4
caryophyllene oxide	1581	0.9
Fonenol	1590	1.8
τ-cadinol	1640	0.8
*cis*-calamenen-10-ol	1661	0.3
Mustakone	1676	0.2
5-epi-paradisiol	1680	0.2
kaur-16-ene	1995	6.5
Cativic acid	-	1.5
3-cleroden-15-oic acid	-	3.6
4-β-kaur-16-en-18-oic acid	-	5.7
Hardwickiic acid	-	8.1
*Terpenoids class*		
*Sesquiterpenes*		60.2
*Diterpenes*		25.4
**Total of identified substances**		85.6

RI = retention index

### Excision wounds

**Fig 1:**
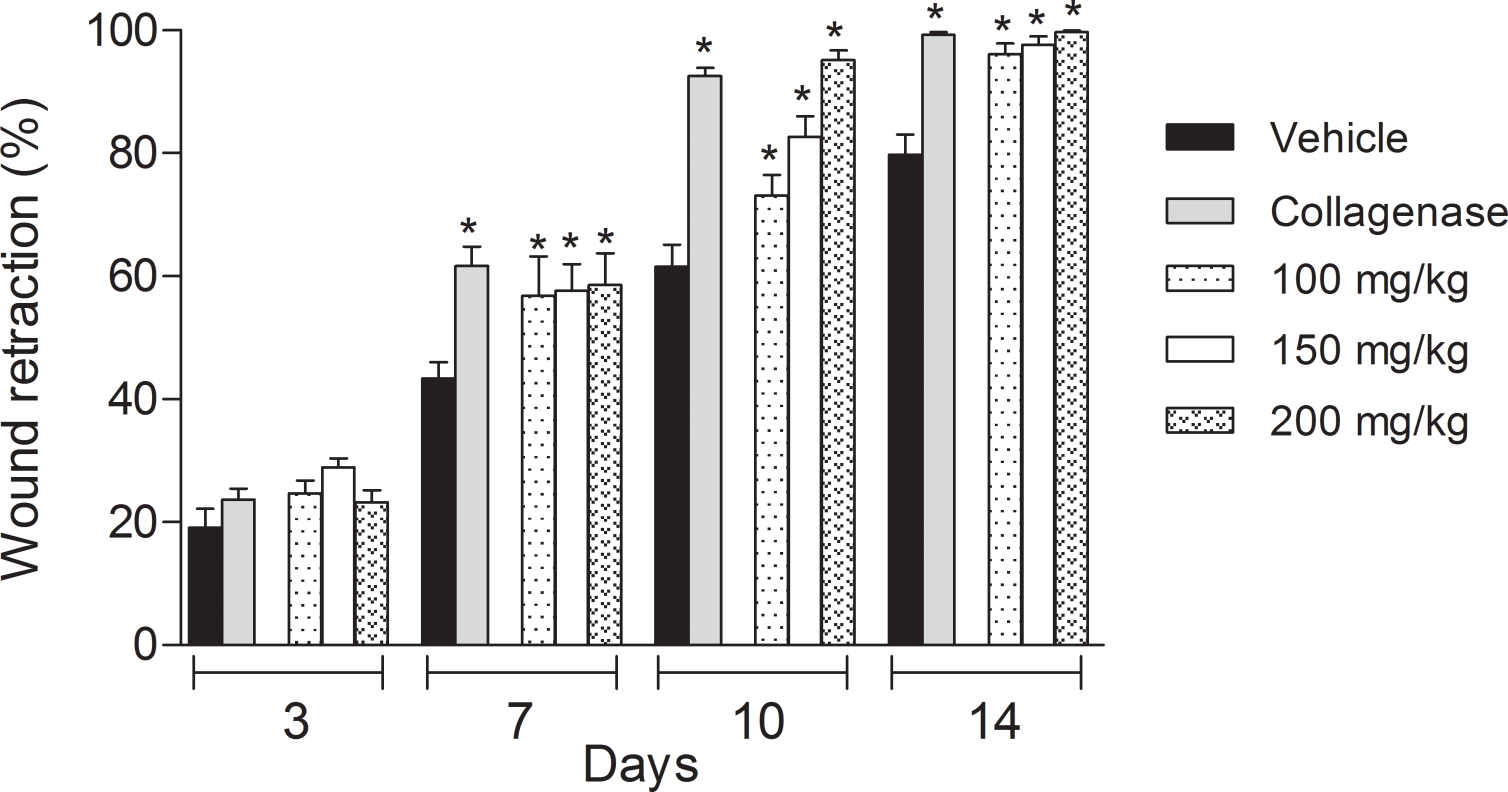
Comparison of wound retraction (in %) between vehicle-, collagenase- and *C*. *paupera* oleoresin-treated diabetic mice (in B) at 3, 7, 10 or 14 days of topical treatment. The results are expressed as media ± standard deviation of wound retraction (%). Statistical analyses were done with two-way ANOVA followed by the Bonferroni post-test. Statistical significance was considered when p < 0.05. * when comparing collagenase-treated mice with collagenase- or *C*. *paupera* oleoresin-treated diabetic mice with vehicle-treated mice (in B).

Development of diabetes in mice resulted in a delay in wound retraction and healing. We observed that 7 days post-wound excision, *naïve* mice (animals without diabetes and without any treatment) showed retracted wounds in 70%, while in diabetic mice, the reduction was 43.4 ± 7.4%. Normal mice showed completely healed wounds at the 14^th^ day (data not shown). However, in the same period, diabetic mice demonstrated retraction of 79.7%. The topical treatment of wounds of diabetic mice with collagenase ointment (at 0.6 U/kg) induced complete wound retraction at day 10 (Fig 1).

Treatment of diabetic mice with any dose of *C*. *paupera* (100, 150 or 200 mg/kg) resulted in an acceleration of the healing process. Seven days after excision and daily topical application with *C*. *paupera* (200 mg/kg), the wounds were retracted in a similar pattern to that observed in the collagenase-treated group. At day 14 post-excision, all oleoresin-treated groups demonstrated an effect similar to the collagenase-treated group (Fig 1).

### Cytokine measurements

**Fig 2:**
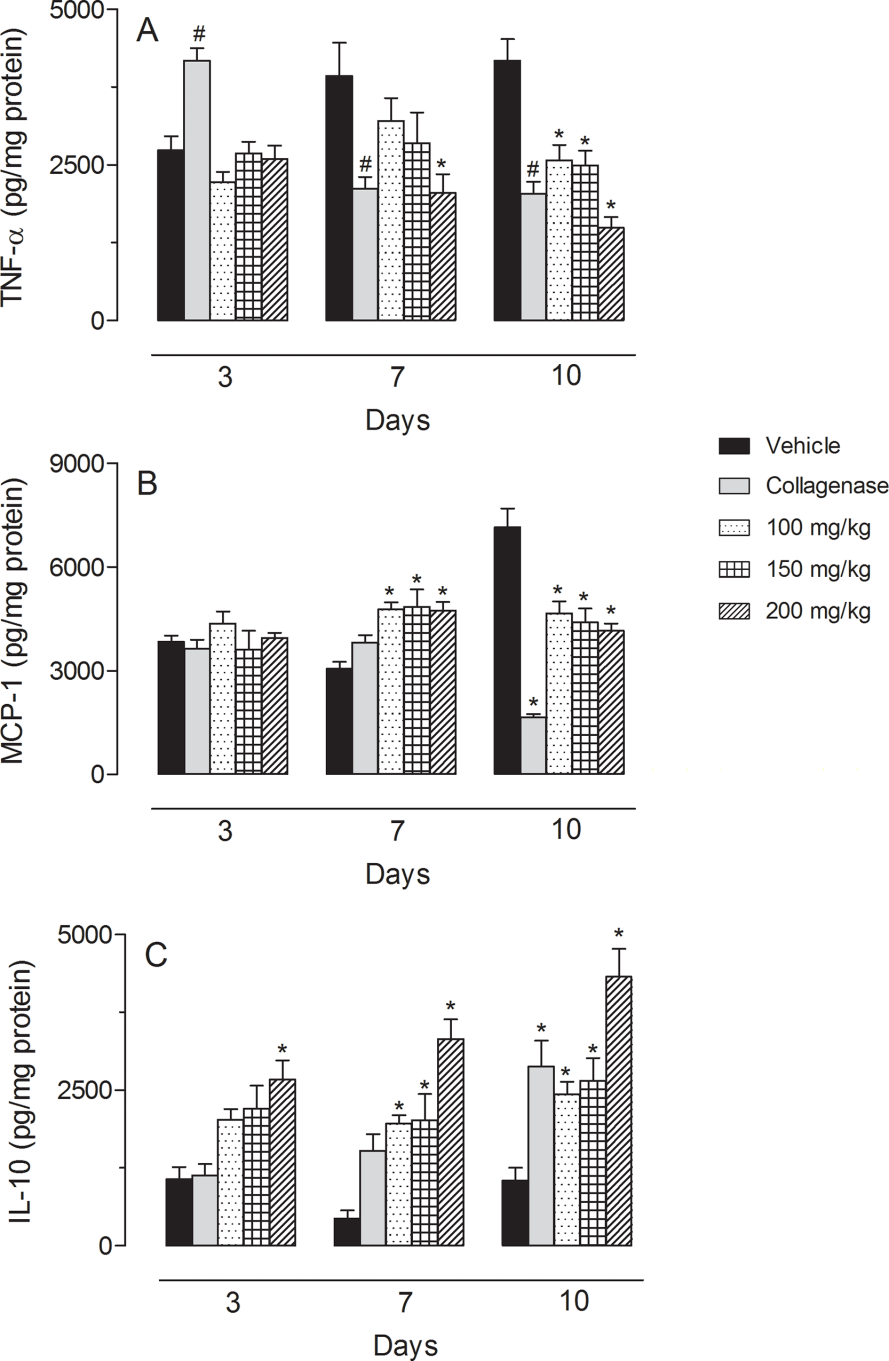
**TNF-α (in A), MCP-1 (in B) and IL-10 (in C) levels diabetic mice after treatment with vehicle, collagenase or *C*. *paupera* oleoresin (100, 150 or 200 mg/kg) for 3, 7 and 10 consecutive days).** Results are reported as median ± standard deviation of pg of cytokine per mg of tissue. Statistical analyses were done with two-way ANOVA followed by the Bonferroni post-test. Statistical significance was considered when p < 0.05. * when comparing collagenase- or *C*. *paupera* oleoresin-treated mice with vehicle-treated mice.

Daily treatment with collagenase did not interfere with the levels of IFN-γ, IL-1-β or IL-6. Similarly, treatment of diabetic mice with *C*. *paupera* also did not reduce the cytokine levels (Data not shown). TNF-α levels were reduced after topical treatment of diabetic mice with collagenase. After 7 consecutive days of treatment with *C*. *paupera* (200 mg/kg), TNF-α levels were significantly reduced. At the 10^th^ day, all doses of *C*. *paupera* significantly reduced the amount of cytokine quantified in the tissue. The treatment also affected MCP-1 levels. At day 7 all doses of *C*. *paupera* increased levels of this cytokine, while at day 10 all doses reduced this cytokine. We also measured the amount of IL-10 accumulated in wound tissues. As we can observe in Fig 2C, even at day 3 post-treatment, the higher dose of oleoresin (200 mg/kg) significantly increased the levels of that cytokine. It is interesting to note that collagenase did not affect IL-10 levels on the 3^rd^ and 7^th^ days. The positive treatment group (collagenase-treated animals) only demonstrated a significant effect on the 10^th^ day. In the same period, oleoresin-treated mice presented an important increase in the amount of IL-10 accumulated in the wound tissues. The amount of IL-10 in diabetic mice after 10 days of treatment with *C*. *paupera* oleoresin was significantly higher than in the collagenase-treated mice (Fig 2A, 2B and 2C).

### Histological evaluation

**Fig 3:**
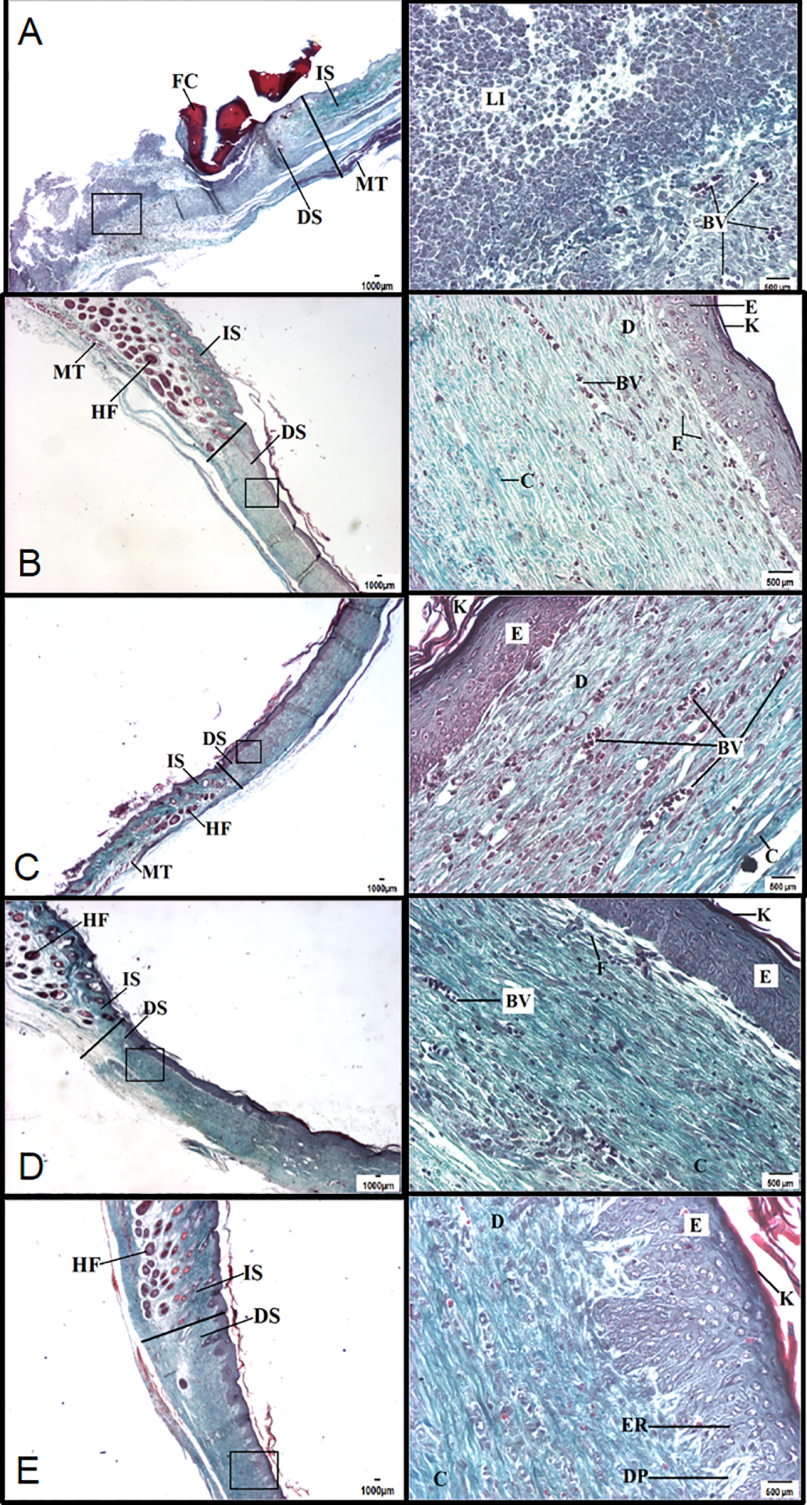
Histological evaluation of wound healing in mice treated with *Copaifera paupera* oleoresin. Mice were topically treated with *C*. *paupera* oleoresin (100, 150 or 200 mg/kg) for 14 consecutive days. At the 14^th^ day, tissues were collected and processed for trichromic Gomori staining. Left panels are visualized intersection areas between normal and lesioned skin at 10X (500 μm). Right panel images were obtained at 20X (500 μm). (A) Vehicle, (B) Collagenase, (C) *C*. *paupera* oleoresin (100 mg/kg), (D) *C*. *paupera* oleoresin (150 mg/kg), (E) *C*. *paupera* oleoresin (200 mg/kg). IS = intact skin; DS = damaged skin; FP = hair follicle; VS = blood vessel; IL = leukocyte infiltrate; E = epidermis; D = dermis; MS = muscle tissue; CF = fibrin-leukocyte crust; F = fibroblast; C = collagen; K = keratin; GS = sebaceous gland; ER = epidermal ridge; DP = dermal papillae.

Significant differences between vehicle and *C*. *paupera*-treated groups were not observed at the 7^th^ day post-excision in diabetic mice. Tissues obtained at the 14^th^ day post-excision and treated with all doses of *C*. *paupera* oleoresin presented a higher area of re-epithelialization when compared with vehicle-treated groups (Fig 3). It is possible to observe a small amount of keratin, and the re-epithelialization was more pronounced with the presence of epidermal ridges and dermal papillae in the group treated with oleoresin (200 mg/kg). Collagen deposition was higher in *C*. *paupera*-treated groups than in the vehicle one. A reduction in inflammatory infiltrate was also observed, whereas vessel numbers were not affected (Fig 3).

**Fig 4:**
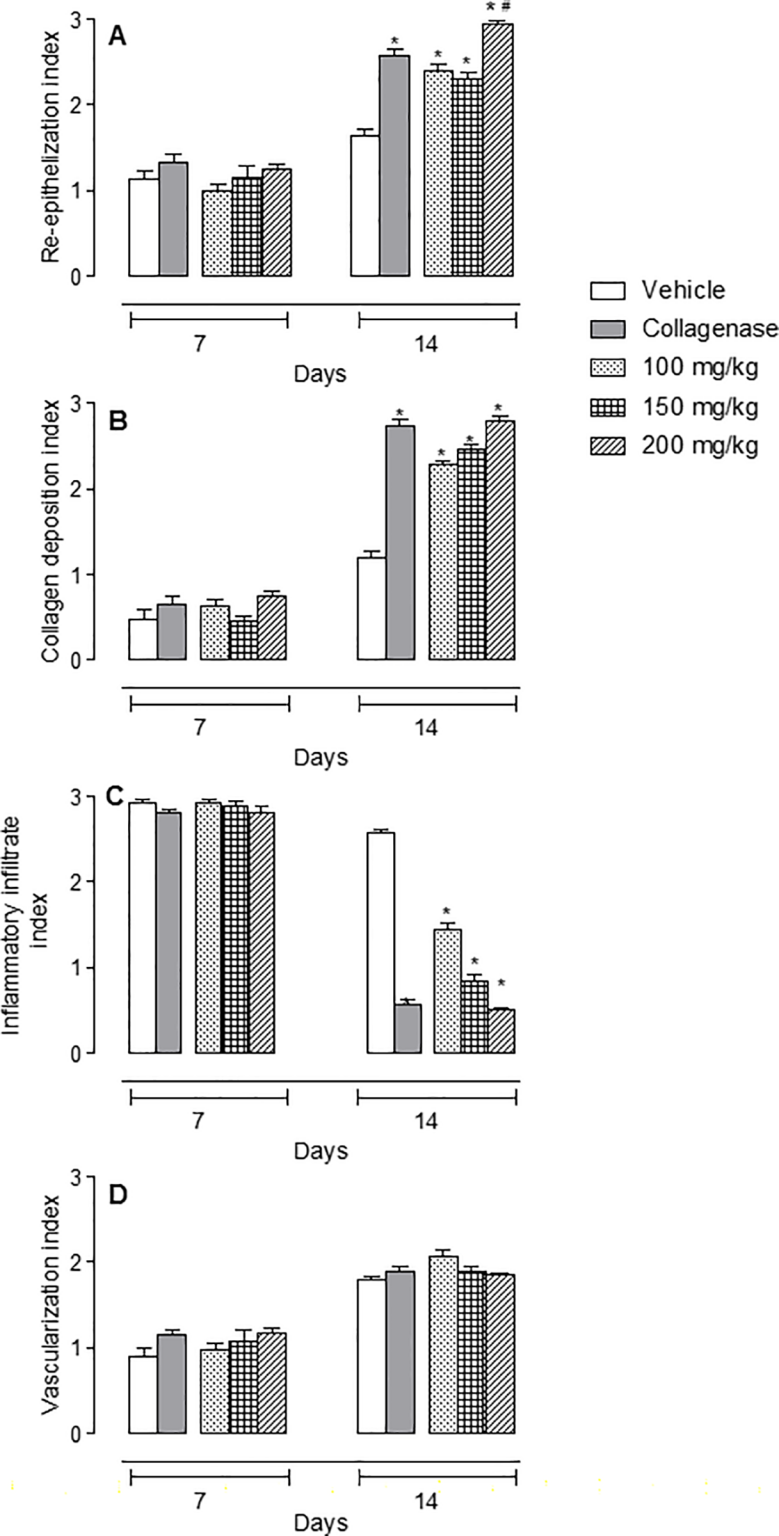
Histological evaluation of wound healing in mice treated with *Copaifera paupera* oleoresin. **Mice were topically treated with *C*. *paupera* oleoresin (100, 150 or 200 mg/kg) for 14 consecutive days**. At days 7 and 14, tissues were collected and processed for trichromic Gomori staining. Re-epithelialization, collagen deposition, inflammatory infiltrate and vascularization index were calculated as described in methods section. The results are expressed as the median ± standard deviation of index. Statistical analyses were calculated with two-way ANOVA followed by the Bonferroni post-test. Statistical significance was considered when p < 0.05. * was considered significant when comparing collagenase- or *C*. *paupera* oleoresin-treated groups with vehicle-treated group and ^#^ was considered significant when comparing *C*. *paupera* oleoresin-treated group with collagenase-treated group.

Histological differences were observed between the groups treated with *C*. *paupera* or collagenase and the vehicle-treated group on the seventh day. The inflammatory infiltrate was classified as abundant in all groups, being present in the surface and deep layers. There was collagen deposition, and skin was observed to be partially re-epithelialized on the edge of the lesion, while in the middle of the lesion, it was not possible to differentiate the layers. There was abundant vascularization in the dermis of all groups. Several differences were observed among the groups on the 14^th^ day. All treated groups presented areas of re-epithelialization. It was also possible to note epidermal layers and the presence of keratin. However, animals treated with *C*. *paupera* (200 mg/kg) presented an increase in re-epithelialization process and formation of epidermal ridges and dermal papillae even when compared with the collagenase-treated group. All *C*. *paupera*-treated mice presented similar levels of increase in collagen deposition and reduction of inflammatory infiltrate when compared with the collagenase-treated group (Figs 3 and 4).

## Discussion

The anti-inflammatory activity of Copaifera oleoresin has been described in many ethnopharmacological studies [3–5, 7], and in addition to its ability to sustain cell proliferation observed in vitro [10], it can contribute to wound healing. However, there are many contradictions among the results described in the literature, probably due to variation in the oleoresin's chemical composition even when obtained from the same species [11, 12]. Additionally, there are some studies that associate the variation in the chemical constituents of copaiba oils with seasonality, soil and region. However, it is known that some *Copaifera* species always have, regardless of these factors, a major constituent. In this context, the species C. langsdorffi and *C*. *martii* can be mentioned as having the main constituent β-caryophyllene, and *C*. *paupera* and *C*. *piresii* present α-copaene as their main component. This fact was observed in this work, where α-copaene was the main terpenoid in the copaiba oil used for the pharmacological test [11, 12].

The chemical composition of the volatile fraction of *C*. *paupera* has been described in the literature [13]. *C*. *paupera* oleoresin has terpenoids with antimicrobial activity. Tincusi et al. [14] isolated twelve diterpenes, three sesquiterpenes and a terpenoid, and tested them against the fungus *Candida albicans* and a few Gram-positive and Gram-negative bacteria. In the present work, the sesquiterpene α-copaene was the major compound in the oleoresin, encompassing 22.9% of the total mass. Zoghbi et al. [13] also observed high α-copaene content (42.5%) in *C*. *paupera* oleoresin.

Wound healing is an important phenomenon in response to tissue injury and involves a series of events that aim to restore structural and functional integrity of the damaged tissue. This process is delayed and reduced in some pathological conditions causing difficulties in healing wound [15].

The healing effect of Copaifera oleoresins is one of the most mentioned activities in ethnopharmacological studies. However, there are several data showing discrepant results. Brito et al. [16] and Vieira et al. [17] showed that oleoresin from *C*. *reticulata* or *C*. *langsdorffii* impaired the wound repair process. Westphal et al. [18] reported an increase in tissue inflammation in rats after intrapleural injection of *C*. *multijuga* oleoresin. Another report related that oleoresin from *C*. *langsdorffii* had no effect on wound healing in intestinal mucosa [19]. This diversity of effects can be attributed to different species from which oleoresin was obtained, leading to a different chemical composition that would inevitably lead to a different pharmacological outcome. It may be that an intense anti-inflammatory action from the previously tested oleoresins could be responsible for the reduced healing effect since an inflammatory infiltrate in wound areas is necessary to start tissue repair and complete the healing activity.

Our results showed an excellent healing effect after 14 consecutive days of topical treatment. These data corroborate previous results from Paiva et al. [20] that showed wound-healing effects of *C*. *langsdorffii* oleoresin in rats. It is difficult to compare all the results due to a number of differences presented. In fact, previous papers used oral administration of oleoresins to treat external or internal (i.e., ulcers) wounds, while our work used topical application of *C*. *paupera* oleoresin.

During the wound healing process, keratinocytes present in epidermal tissue release IL-6, IL-1β and TNF-α, cytokines that are important as chemoattractants for cells [21]. IL-1β is a cytokine responsible for stimulating the expression of adhesion molecules on endothelial cells, allowing the infiltration of immune cells present in the blood to injured tissue [22]. This fact is important to the start of the tissue repair process, especially in the inflammatory phase, which requires defense cells to prevent possible infections. According to Ram et al. [23], a high and persistent level of this cytokine is responsible for a delay in the formation of granulation tissue, resulting in the failure of wound closure. Thus, the decrease observed in IL-1β levels in the group treated with *C*. *paupera* may be one of the factors responsible for the significant contraction of the lesions.

Increased IL-10 production can lead to a decrease in generation of inflammatory mediators, producing a better environment for the wound healing process in skin with less scar formation [24]. It is possible that the decrease in IL-1β levels observed in our work is due to an IL-10 increase and that the high levels of IL-10 in groups treated with the *C*. *paupera* improved the organization of the collagen fibers.

Our data also showed that after treatment with collagenase ointment, TNF-α levels increased at the third day and were reduced at the seventh and tenth days when compared to the control. They also indicate that *C*. *paupera* treatment reduced TNF-α levels in the skin. This can justify the improvement observed in the treated groups, suggesting that there was a decrease in the inflammatory phase duration, supporting the evolution of the wound to the proliferative and later stages of contraction of the lesion.

Collagen is the most abundant connective tissue protein in the healing process. The normal dermis has approximately 80% of type I collagen and 20% type III collagen. Since the granulation tissue expresses 30 to 40% type III collagen, it is considered immature collagen. The increase in mature collagen is directly proportional to the tensile strength of the wound, and tissue remodeling is closely related to the deposition of type III collagen, which is gradually replaced by type I collagen [25]. This substitution can be observed when comparing the histological findings on the 14^th^ day. The treated animals showed more mature collagen fibers. Therefore, the histological data suggest that treatment of skin lesions with the oleoresin accelerated formation of type I collagen, accelerated the healing process and improved the quality of scar tissue. Another interesting finding was that oleoresin-treated mice showed epidermal ridges and reconstructed dermal papilla in the area of the lesion. Thus, it is possible that the process of healing is faster after treatment with the *C*. *paupera* oleoresin even when compared with the collagenase-treated group.

It is notable that on the 14^th^ day, the vehicle-treated mice exhibit a pronounced leukocyte infiltration, characterized by persistence of the inflammatory phase and formation of granulation tissue, while those groups that received collagenase or *C*. *paupera* oleoresin treatment showed a slight leukocyte infiltration. The results obtained so far suggest that a reduction of the inflammatory response in the treated groups, as evidenced by decreased levels of pro-inflammatory cytokines, increased levels of IL-10 and decreased leukocyte infiltration, as well as the increase in collagen deposition and stimulation of migration and proliferation of keratinocytes at the wound edge all contribute to accelerate the healing process.

## Conclusions

Our data indicate that *C*. *paupera* oleoresin presents high healing potential, facilitating the regenerative process and reducing the healing time, which may place it as a candidate for the topical treatment of cutaneous lesions. Taken together, these results allow us to state the benefits of this Copaifera oleoresin as a wound-healing product, justifying its traditional use.
